# When does repetition suppression depend on repetition probability?

**DOI:** 10.3389/fnhum.2014.00685

**Published:** 2014-09-02

**Authors:** Gyula Kovács, Rufin Vogels

**Affiliations:** ^1^Institute of Psychology, Friedrich-Schiller University of JenaJena, Germany; ^2^Department of Cognitive Science, Budapest University of Technology and EconomicsBudapest, Hungary; ^3^Laboratorium voor Neuro- en Psychofysiologie, Onderzoeksgroep Neurofysiologie, Departement Neurowetenschappen, KU Leuven Medical SchoolLeuven, Belgium

**Keywords:** fMRI, predictive coding, repetition suppression, adaptation, objects, attention

It has been shown recently that the probability of stimulus repetitions (P_rep_) determines the degree of repetition suppression (RS): the repetition-related decrease of the fMRI signal (fMRI adaptation, fMRIa) was larger for faces in the fusiform face area (FFA) in blocks with high when compared to low repetition probabilities (Summerfield et al., 2008). This suggests that higher-order contextual expectations, via top-down connections, modulate fMRIa and the results were interpreted in the frame of predictive coding models (Rao and Ballard, 1999; Friston, 2005). This effect was confirmed for face stimuli in independent laboratories (Kovács et al., 2012; Larsson and Smith, 2012). However, the generality of the P_rep_ effect on RS was questioned recently, using non-face stimuli. First, Kaliukhovich and Vogels (2010) observed no P_rep_ modulation of the RS of the spiking activity and local field potentials in awake behaving rhesus monkeys for fractal patterns and everyday objects. Second, using stimulus-sets (everyday objects and chairs) that partially overlapped the monkey study, Kovács et al. (2013) also found no P_rep_ modulations of the fMRIa in LO, a proposed homolog of the macaque IT. This suggested that prediction effects may vary across visual categories and this variance could be due to differences in selectivity for faces and objects in face and object selective areas respectively, an idea requiring further testing. Furthermore, current data underlines the role of prior experience in generating P_rep_ modulations (Grotheer and Kovács, 2014).

More recently, Mayrhauser et al. (2014) employed line-drawings of objects to test P_rep_ effects in LO. Contrary to Kovács et al. (2013), they found P_rep_ modulations of RS for line-drawings in the left LO. They argued that the reason of the discrepancy between the studies is the “*diverging extent of repetition probability*,” claiming that expectations were manipulated in the Kovács et al. (2013) study using 60/20% of repetition trials in repetition/alternation blocks, respectively, while in their and prior investigations using faces, P_rep_ varied between 75/25%. They argued that the less extreme range of P_rep_ in the Kovács et al. (2013) study could “*underrun a critical difference which is needed to reliably indicate modulatory effects of perception expectations for objects*.” However, as detailed in the methods section of the Kovács et al. (2013) paper, that study has in fact used even stronger differences as compared to the Summerfield et al. (2008) study. Figure 1B in our previous paper states that a block contained 20% target trials and 80% non-target trials with 60 and 20% frequent and rare trial types, respectively. (Please note that this equals to 75%/25% proportions for the non-target trials, identical to previous studies). In addition, in our study the target trials consisted of alternation or repetition trials with the same probabilities as the non-target trials (leading to an overall 80%/20% proportion within an entire block).

**Figure 1:**
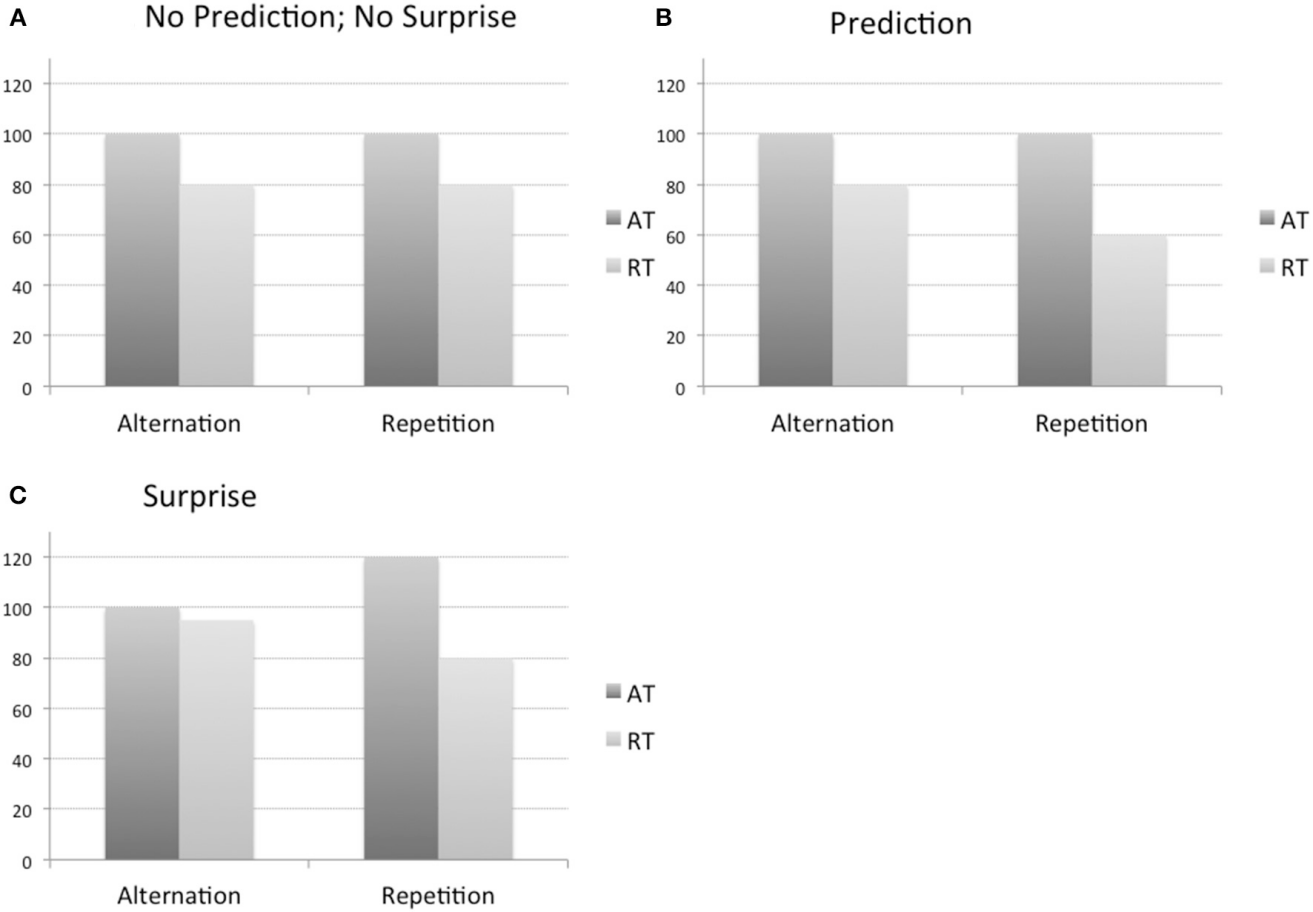
**Schematic illustrations of how top-down modulations (predictions and surprise-related) might affect neural responses**. Hypothetical fMRI response is depicted separately for the blocks with low probability of repetitions (Alternation) and high probability of repetitions (Repetition). **(A)** Repetition suppression is independent of predictions or surprise. **(B)** Repetition suppression is enhanced for expected, repeated stimuli. **(C)** Responses are enhanced for surprising stimuli. Results for face stimuli (FFA, OFA), objects (macaque IT and LO), outlines of objects (left and right LO), and roman letters (LFA, LO) are mentioned only. FFA, fusiform face area; IT, inferior temporal cortex; LFA, letter form area; LO, lateral occipital cortex; OFA, occipital face area; AT, alternation trial; RT, repetition trial. Cortical areas, where the responses were in accordance with the three possibilities are the following: **(A):** IT (Kaliukhovich and Vogels, 2010); LO (Kovács et al., 2013); right LO (Mayrhauser et al., 2014). **(B):** FFA (Summerfield et al., 2008 E1); LO1 (Larsson and Smith, 2012); FFA (Kovács et al., 2013). **(C):** FFA (Summerfield et al., 2008 E2, Kovács et al., 2012, Larsson and Smith, 2012); OFA (Kovács et al., 2012); LO (Kovács et al., 2012; Grotheer and Kovács, 2014); LFA (Grotheer and Kovács, 2014); left LO (Mayrhauser et al., 2014).

Thus, it is impossible that the discrepancy between the Kovács et al. (2013) study and the previous studies using faces and line drawings is caused by a smaller difference in repetition probability in the latter study.

What then could have caused the discrepancy between the studies? Mayrhauser et al. (2014) observed P_rep_ effects only in the left hemisphere (LH). In the right hemisphere (RH) they observed RS in both high and low repetition-probability blocks and, importantly, the magnitude of this RS was similar between the blocks. Thus their RH LO data fully agrees with that of the Kovács et al. (2013) paper [please note that the location of LO is slightly more superior in the Kovács et al. (2013) when compared to the Mayrhauser et al. (2014) study for both hemispheres and, although unlikely, this might explain some differences of the two studies over the LH]. Interestingly, Mayrhauser et al. (2014) did not observe RS in the LH in the low-probability blocks, which is similar to what was recently observed for Roman letters in LO (Grotheer and Kovács, 2014). In fact, a closer inspection of the Mayrhauser et al. (2014) and Grotheer and Kovács (2014) data suggests that the P_rep_ effect in both studies is due to an enhanced BOLD signal in alternation compared to repetition trials in high-probability blocks (Figure 1C). This suggests that the alternation trials in the block with a high number of repetition trials leads to an enhanced response to that rare, surprising event, which then produces a P_rep_ effect. Given that a P_rep_ effect may only manifest itself when attention is directed to the stimuli (Larsson and Smith, 2012) only familiar stimuli that automatically engage attention such as faces (and perhaps letters) may show the surprise effect. Another possibility is that the surprise response is only present for highly familiar, well-represented objects, allowing highly specific expectations. The reason why such a surprise related P_rep_ was present in (only) the LH in the Mayrhauser et al. (2014) study is unclear, one possibility being that the subjects were verbalizing the abstract line drawing stimuli in that study.

Figure 1 summarizes the current findings on the modulation of RS by expectation: either no effect (Figure 1A), a decrease of the response for expected stimuli (Figure 1B) or an increase of the response to the unexpected stimuli (Figure 1C). We propose that RS largely reflects bottom-up or local adaptation mechanisms (Kaliukhovich and Vogels, 2010). This can explain why RS can be observed without any modulation by repetition probability (Figure 1A). However, under some conditions the adaptation effects can indeed interact with expectation/surprise related top-down modulations, explaining the P_rep_ effects observed in some studies (Figures 1B,C), which can range from an increased suppression for repeated, expected stimuli to an enhanced response to unexpected, deviants. We would like to note that in fMRI studies to date that found an effect of repetition probability the surprise enhancement effect (Figure 1C) appears to be observed more frequently than the expectation suppression (Figure 1B). Defining the conditions under which these top-down modulations operate is the subject of further research. It could be helpful to include in this research a “neutral” condition in which the probabilities for repetitions and alternation trials are equated so that suppressing versus enhancing modulations can be identified unambiguously.

## Conflict of interest statement

The authors declare that the research was conducted in the absence of any commercial or financial relationships that could be construed as a potential conflict of interest.
